# Impaired Spatial Memory after Ketamine Administration in Chronic Low Doses

**DOI:** 10.2174/157015911795016912

**Published:** 2011-03

**Authors:** C Venâncio, A Magalhães, L Antunes, T Summavielle

**Affiliations:** a Instituto de Biologia Molecular e Celular (IBMC), Universidade do Porto, Porto, Portugal; b CECAV, Universidade de Trás-os-Montes e Alto Douro, Vila Real, Portugal

**Keywords:** Ketamine, spatial recognition, memory, habituation, long-term effects.

## Abstract

Ketamine is a noncompetitive antagonist of the NMDA-receptors, used as a dissociative anesthetic, presently included in the category of the psychoactive substances known as “club drugs”. Ketamine administration was associated with impaired working memory and increased psychopathological symptoms, but there is a lack of information regarding the effects of chronic sub-anesthetic doses. Adult Wistar rats were administered ketamine, 5 and 10 mg/kg twice daily, subcutaneously for 14 days. One week later, rats were tested in an object recognition/object location task and in the open field arena. There was altered performance in both the object recognition/location and in the open field tests by the group chronically exposed to the lower dose of ketamine. These animals displayed a decreased discrimination index (p<0.05) in the object recognition task, were unable to recognize the displacement of a familiar object and displayed decreased activity across open filed sessions. Importantly, these alterations were not observed in animals administered a higher dose of ketamine. Collectively, these results consistently show that chronic administration of ketamine in sub-anesthetic doses may lead to decreased habituation and inability to update spatial representations.

## INTRODUCTION

Ketamine is a clinically and veterinary important drug commonly used in anesthesia and perioperative analgesia [1, 2]. It’s psychedelic properties make it also a popular drug of abuse [3]. At subanesthetic doses, the role of ketamine, as a noncompetitive *N*-methyl-D-aspartate (NMDA) receptor antagonist, in blocking the processing of nociceptive inputs has led to its use in chronic pain syndromes management [4, 5]. However, ketamine use, both in preclinical studies [6] or in compulsive users was reported to induce cognitive impairments [7].

Acute subanesthetic doses of ketamine were shown to induce a marked increase in glutamate release in the nucleus accumbens facilitating synaptic flow of information from the prefrontal cortex (PFC) and amygdala [8], which is consistent with the hypothesis that ketamine acts preferentially to block NMDA receptors on inhibitory neurons leading to a state of disinhibition and increased glutamate release in the PFC and limbic regions [8, 9]. Importantly, the administration of chronic and sub-chronic doses of other NMDA antagonists lead to disruption of the hippocampal and PFC function [10-12], Particularly, chronic NMDA-antagonism, has been reported to induce persistent deficits in working memory [10, 13, 14].

With regards to the chronic action of ketamine, inconsistent results have been reported, which seem to reflect different treatment protocols and evaluation schedules. Therefore, the purpose of the present study was to contribute to the clarification of the long term effects of chronic analgesic doses of ketamine in memory disruption. To achieve this goal the object recognition test and object location task were selected to assess, respectively, spatial and non-spatial recognition memory. Locomotor activity and the ability to habituate to a new environment were also monitored.

## MATERIALS AND METHODS

### Animals

Male Wistar rats (colony of the Institute of Molecular and Cell Biology, University of Porto, Portugal) aged 80 to 90 days were used. Rats were housed in pairs in a controlled environment (20±2ºC, 45-55% humidity) with 12-h light/dark cycle. Food and water were supplied “ad libitum”. All experiments were approved by the Portuguese Agency for Animal Welfare (general board of Veterinary Medicine in compliance with the Institutional Guidelines and the European Convention).

Rats were divided in 3 experimental groups as follows: ketamine 5 mg/kg every 12 hours (K 5mg), ketamine 10 mg/kg every 12 hours (K 10mg) and saline or control (vehicle Nacl 9%) in the same administration protocol. The selected doses were shown to be analgesic in previous studies [15, 16]. Ketamine (Imalgene 1000, Merial Portuguesa) was administered subcutaneously (s.c.), in 12 h intervals, in a volume of 1 ml/kg of body weight, for 14 consecutive days. All behavioral experiments were performed during the dark period of light cycle and video recorded. Behavioural data were subsequently analysed using the software Observer XT (Noldus Information Technology, Wageningen, Netherlands).

The object recognition test was performed as describe previously [17]. Briefly, the test apparatus consisted in a grey open box made of PVC (60 x 80 x 40 cm).The objects used were made of plastic, glass or metal in three different shapes: cubes, pyramids and cylinders. Twenty four hours after the last administration of ketamine, rats were allowed to explore the apparatus, for 10 min, with a single object (familiar object) placed in the center of the open box. This was repeated for 5 consecutive days. The object recognition test began after the habituation period. Initially, a sample phase with two identical objects placed in two opposite corners of apparatus (10 cm from the side wall) was utilized. The rat was placed in the middle of the apparatus, allowed to explore these objects for 3 min and then returned to its home cage. Choice phases were conducted with both a delay of 15 min and a delay of 24 hours. The rat was reintroduced into the open box and a choice phase took place for further 3 min. In the choice phase two objects were exposed in the same locations that were occupied by objects in the previous sample phase, one of the objects was identical to those presented in the sample phase and the other was a novel object. The time spent exploring each object was recorded. Exploration was defined as follows: rat touches the object with its nose or the rat’s nose is directed toward the object at a distance shorter than 2 cm. Circling or sitting on the object were not considered as exploratory behaviors. The index of discrimination was calculated as the difference between contact time with the novel and the familiar object [17].

Object location testing was conducted 24 h after the object recognition test. This test is similar to the object recognition task described above, except for the choice phase test, where the two identical objects are re-exposed, but one of them is moved to a novel location. In the object location task, only a delay of 15 min was evaluated.

One week after the last ketamine administration locomotor activity was evaluated by placing a single rat into an acrylic cubic open field arena (40 x 40 x 40 cm), equipped with two parallel arrays of photocells (San Diego Instruments, San Diego, CA). Data were collected in 1 min intervals over 10 minute’s sessions. Each rat was tested for three consecutive days. The following parameters were automatically registered: rearing, central activity, peripheral activity (i.e. frequency of locomotion along the walls of the open-field). Habituation to the open-field environment was also studied by comparing the total locomotor activity across open-field sessions.

Data were analysed by repeated-measures analysis of variance (ANOVA). Whenever significant differences were detected, further comparisons were made using the Student`s *t*-test. In addition, Paired-samples *t*-test was performed on individual groups to examine whether animals demonstrated preference for novelty. Habituation between sessions was also analyzed by Paired-samples *t*–test. The statistical level of significance was considered at p<0.05. Statistic analyses were performed using the software SPSS Statistics 17 (SPSS Inc., IL).

## RESULTS AND DISCUSSION

Body weight gain was monitored throughout the experimental period. A chronic ketamine administration at the doses of 5 (K 5mg) and 10 mg/kg (K 10mg) decreased the evolution of body weight as shown in “Fig. (**1**)”. Two days after the onset of the experimental period, weight gain values in the ketamine treated groups were significantly reduced. This effect persisted after the end of ketamine administration period and throughout the behavioural testing phase. Interestingly, these data are not in accordance with previous reports where a chronic administration (7 days) of ketamine 15 mg/kg (i.p) was shown to increase body weight and sweet food consumption [18]. However, humans chronically administered with low doses of ketamine reported side effects such as nausea and vomiting with consequent loss of appetite [19]. Moreover, ketamine is known to interfere with gustatory trace in a dose dependent manner [20], which can also account for reduced food intake.

Results obtained in the object recognition task show that all experimental groups preferentially explored the unfamiliar object, either when the new object was presented 15 min after the presentation session, or when it was presented with a delay of 24h. These results are shown in “Fig. (**2**)”. Regarding the discrimination based on the duration of exploration, all the groups presented a positive discrimination index between the familiar and unfamiliar object both in the short-term and long-term delay, since all of them spent more time exploring the unfamiliar object “Fig. (**3**)”. However, when discrimination was evaluated based on the time spent exploring the unfamiliar object, the animals treated with the lower dose of ketamine displayed a decreased discrimination index (p<0.05). Interestingly, this seems to be caused by increased exploration of the familiar object and to some extent may reflect altered habituation properties. Habituation is a complex function; however, the relevance of the hippocampus in this process is well established. In rodents, habituation may be seen as a decreased exploratory behaviour in response to continued or repeated environment or stimulus [21]. Furthermore, habituation is commonly used for assessment of non-associative learning and memory processes. Glutamate is known to facilitate memory retention, while NMDA antagonists were shown to decrease habituation in a dose dependent manner [22]. It was reported that MK-801 administration did not interfere with the ability to recognize a novel object but prevented the correct identification of spatial changes, suggesting that NMDA blockade may result in inability to update spatial representations [23].

Importantly, in the object location task, while the control animals and the group treated with a higher dose of ketamine (10 mg K) were able to distinguish between a familiar object in a familiar location, and an equal object in a novel location (p<0.01 and p<0.05, respectively); the animals treated with a lower dose of ketamine (5 mg K) were unable recognize the displacement “Fig. (**4**)”. These results reinforce the inability presented by the animals chronically treated with 5 mg of ketamine, to display an adequate habituation, which can result from altered glutamatergic function. Spatial processing has been shown to relate with infra pyramidal mossy fibber terminals that form glutamatergic synaptic contacts on complex branching spines [24]. Although, at the synaptic level, electrophysiological and imaging studies suggested a stronger involvement of AMPA receptors than NMDA receptors [25, 26], it was shown that blocking both types of receptors would alter the formation of clusters at the terminal level, which would translate into altered hippocampal function [27].

In the open field, while total or locomotor activity was not affected by ketamine, the group treated with 5 mg of ketamine failed to reduce activity across sessions “Fig. (**5**)”. In the open field, rodents habituation was evaluated by declined activity through consecutive daily sessions [21]. In the present work, only controls and animals treated with 10 mg of ketamine displayed this adaptation (p<0.05 and p<0.001, respectively). In accordance with what was observed in the object recognition/location task, these results also point towards impaired habituation in animals chronically administrated with 5 mg of ketamine and may be a consequence of disrupted spatial memory. This is in agreement with previous reports were a single administration of very low doses of ketamine (1 and 3 mg /Kg) was shown to impair spatial memory in an object location test [28]. Recently, in rats treated for 10 days with 30 mg/Kg of ketamine (twice a day) and tested in a radial maze 10 to 20 days after cessation of treatment, a persistent impairment of spatial working memory was observed and associated to altered function of the prefrontal cortex but not the hippocampus [14]. However, in that study, rats were trained before exposure to ketamine and therefore acquisition and consolidation of spatial/contextual information was achieved prior to NMDA antagonism.

Another interesting aspect of the present results is the consistent difference observed between the effects of a 5 mg/Kg dose and a 10 mg/Kg dose. Although the molecular mechanisms that underlie the present results remain unclear, a previous study, that investigated the dose-response characteristics of ketamine, has shown that a 4 mg/Kg dose was able to induce a much higher expression of c-fos in the hippocampus and amygdalar regions, than doses of 8, 12 or 16 mg/Kg [6]. It was proposed that this dose-effect could be due to the indirect activation of excitatory neurotransmitter systems, as a consequence of reduced activity of GABAergic neurons under low doses of NMDA antagonists [6]. Importantly, the glutamatergic afferents from the hippocampus and the amygdala provide a gating of information flow into the prefrontal cortex [29] and increased expression of c-fos in these regions is likely to be associated with altered cognitive function.

In summary, viewed collectively, the present results provide consistent evidence of inability to update spatial representations and decreased habituation properties, induced by chronic treatment with sub-anesthetic doses of ketamine. This is particularly relevant for the use of ketamine in the analgesia in chronic-pain syndromes.

## Figures and Tables

**Fig. (1):**
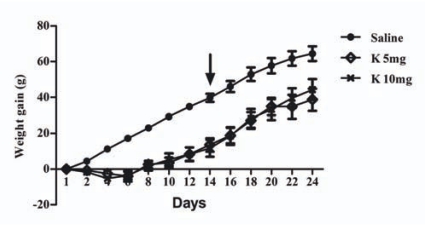
Decreased body weight gain. Weight gain throughout the experimental period for the three groups of rats under study: saline (n=18), K 5mg (ketamine 5 mg/kg, n=17) and K 10mg (ketamine 10 mg/kg, n=17). Each point represents the mean ± standard error of body weight in a given day. Since day 3, animals treated with either dose of ketamine displayed significantly decreased weight gain as determined by the Turkey HSD for unequal N test. The arrow indicates the end of the administration period.

**Fig. (2):**
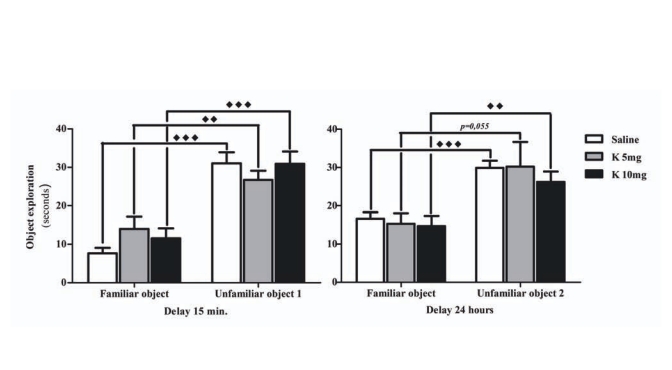
Time spent exploring the familiar and unfamiliar objects in the choice phase of the object recognition task, using both a 15 min and a 24 h delay. Male Wistar rats were treated with 5 mg/kg of ketamine (K 5mg, n=9) and 10 mg/kg of ketamine (K 10mg, n=9), in 12 h intervals. The control group received an isovolumetric dose of saline solution following the same protocol (Saline, n=10). Results were represented as the mean + standard error for each group (expressed as time in sec). Significant differences were signed as ♦♦*p*<0.01 or ♦♦♦*p*<0.001.

**Fig. (3):**
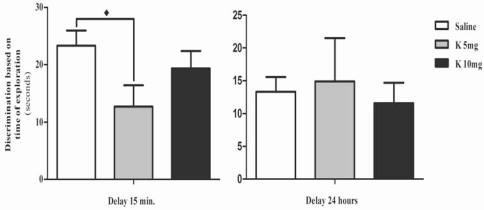
Effects of chronic ketamine administration on discrimination index performance, determined as the difference between the time spent exploring the novel object and the familiar, after both a 15 min and a 24 h delay. Male Wistar rats were treated with 5 mg/kg of ketamine (K 5mg, n=9) and 10mg/kg of ketamine (K 10mg, n=9), in 12 h intervals. The control group received an isovolumetric dose of saline solution following the same protocol (Saline, n=10). Results were represented as the mean + standard error for each group (expressed as time in sec). Significant differences were signed as ♦ *p*<0.05.

**Fig. (4):**
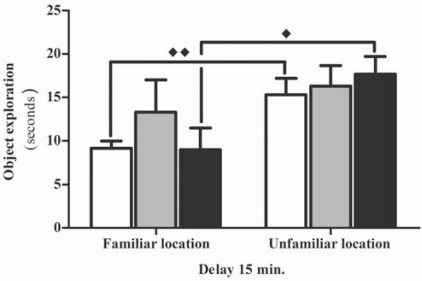
Time spent exploring objects placed in familiar or unfamiliar locations in the choice phase of the object location task, using a 15 min delay. Male Wistar rats were treated with 5 mg/kg of ketamine (K 5mg, n=9) and 10mg/kg of ketamine (K 10mg, n=9), in 12 h intervals. The control group received an isovolumetric dose of saline solution following the same protocol (Saline, n=10). Results were represented as the mean ± standard error for each group (expressed as time in seconds). Significant differences were signed as ♦*p*<0.05 or ♦♦ *p*<0.01.

**Fig. (5):**
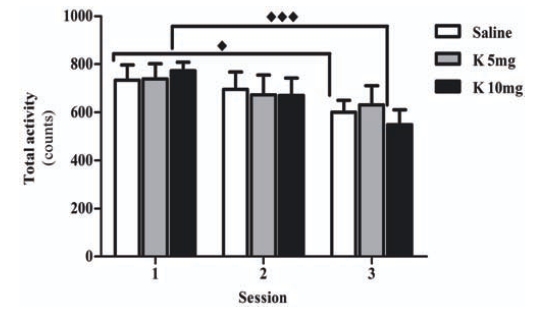
Effects of chronic administration of ketamine on total activity and habituation into the open-field arena. Male Wistar rats were treated with 5 mg/kg of ketamine and 10mg/kg of ketamine (K 10mg, n=9), in 12 h intervals. The control group received an isovolumetric dose of saline solution following the same protocol (Saline, n=10). The results were represented as the mean ± standard error (expressed as counts in 10 min sessions). Significant differences were signed as ♦*p*<0.05 or ♦♦♦*p*<0.001.
